# Supplementary figures and images for: Low-carbon optimal scheduling of integrated energy systems based on multi-strategy ameliorated goose algorithm and green certificate-carbon trading coordination
Source: PLoS One. 2025 Sep 12;20(9):e0331927. doi: 10.1371/journal.pone.0331927 (PMC12431214; doi:10.1371/journal.pone.0331927)

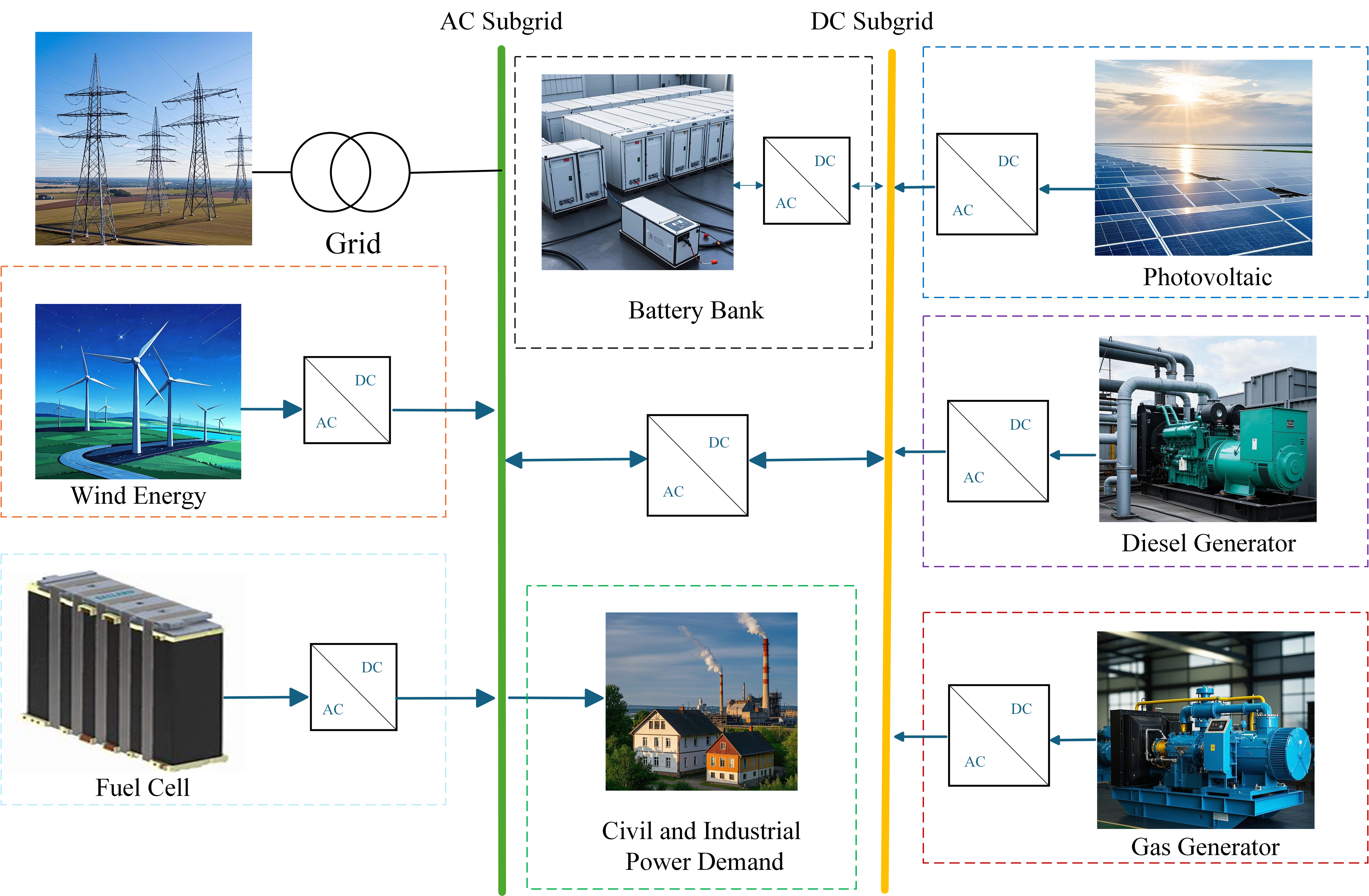

Supplement: S1 Fig — (ZIP) [file pone.0331927.s001.zip › S1 Fig/Fig1.tif]

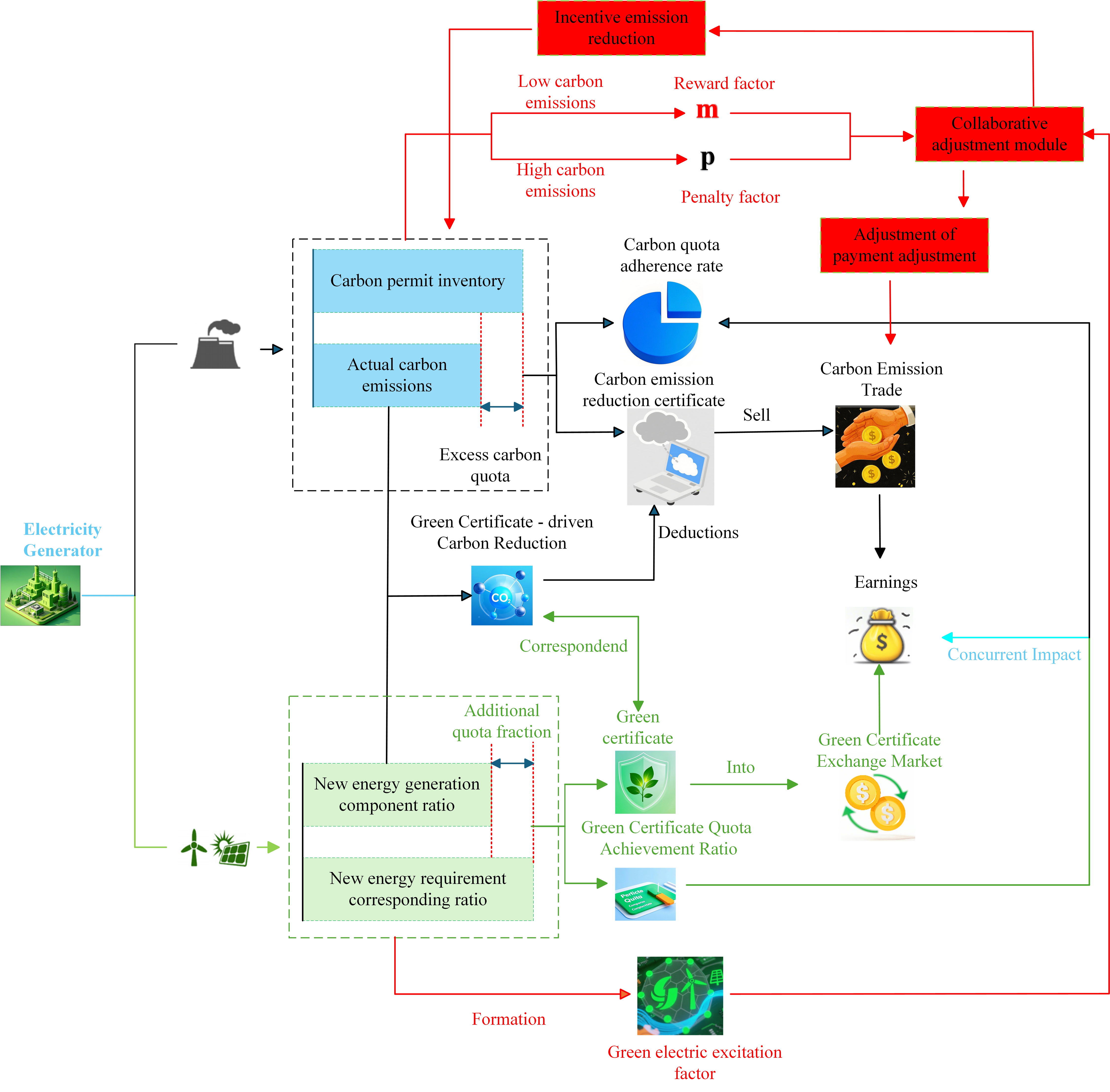

Supplement: S1 Fig — (ZIP) [file pone.0331927.s001.zip › S1 Fig/Fig2.tif]

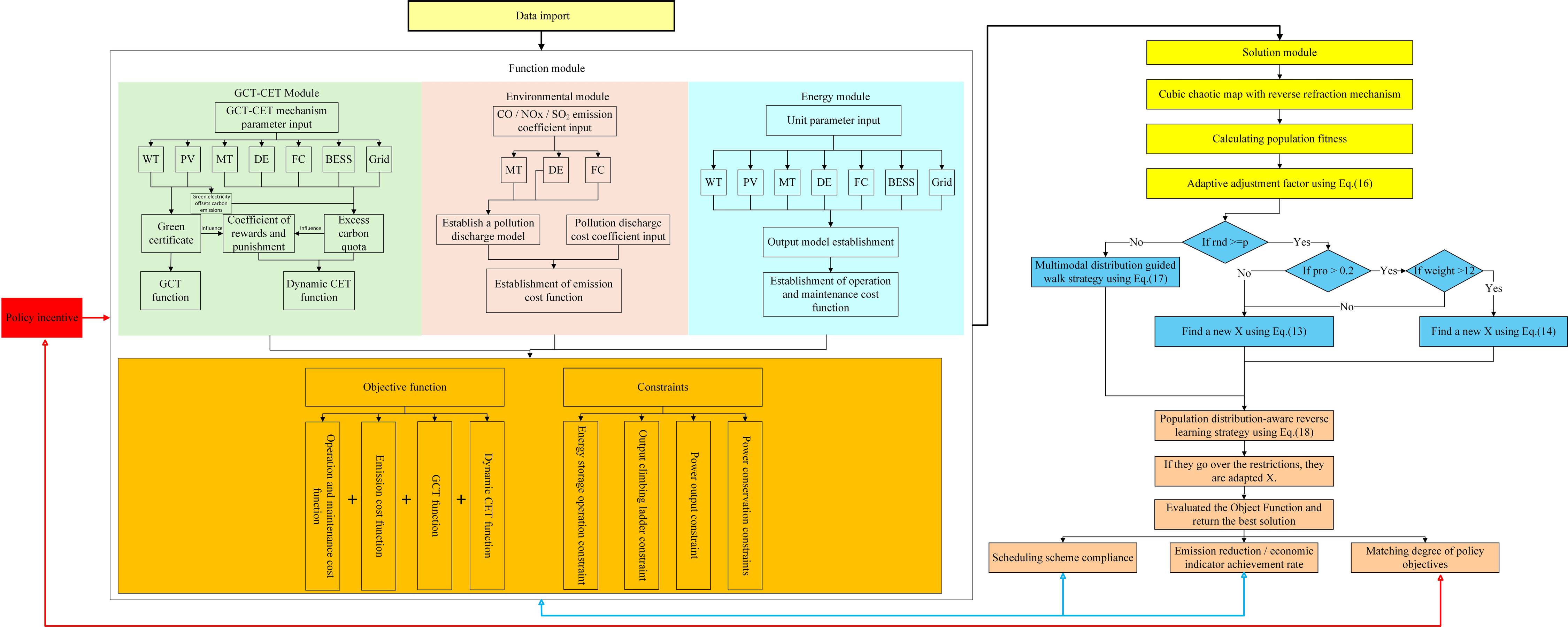

Supplement: S1 Fig — (ZIP) [file pone.0331927.s001.zip › S1 Fig/Fig3.tif]

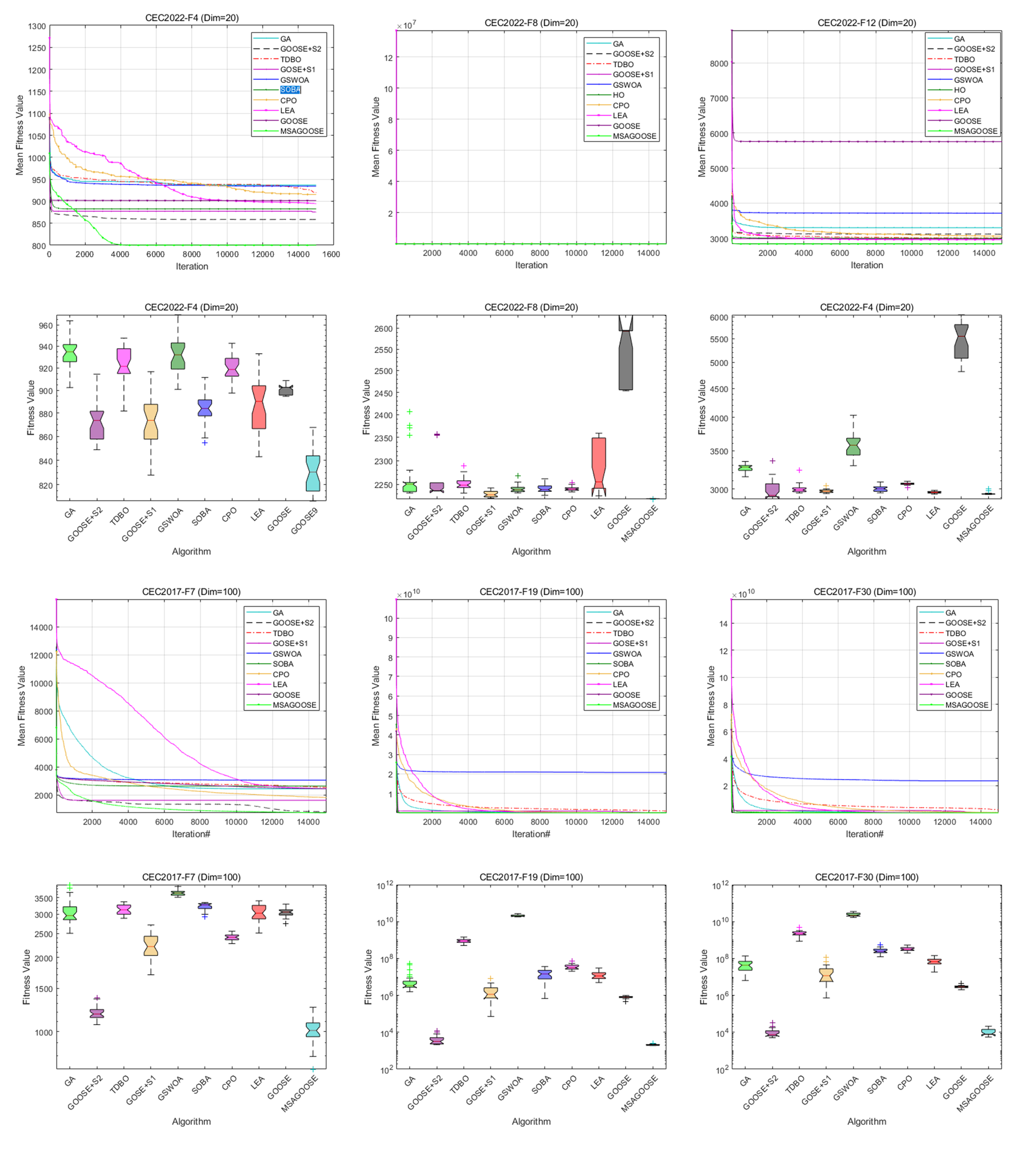

Supplement: S1 Fig — (ZIP) [file pone.0331927.s001.zip › S1 Fig/Fig4.tif]

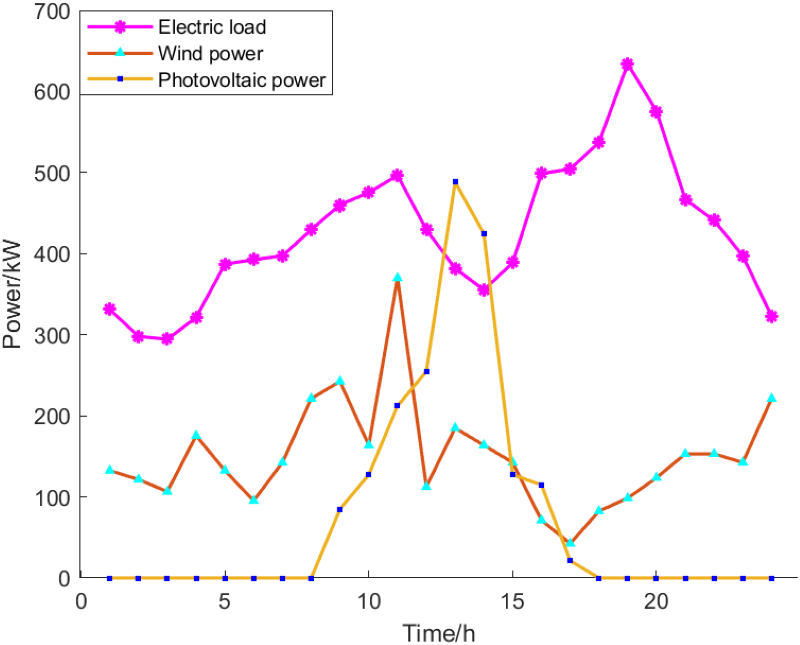

Supplement: S1 Fig — (ZIP) [file pone.0331927.s001.zip › S1 Fig/Fig5.tif]

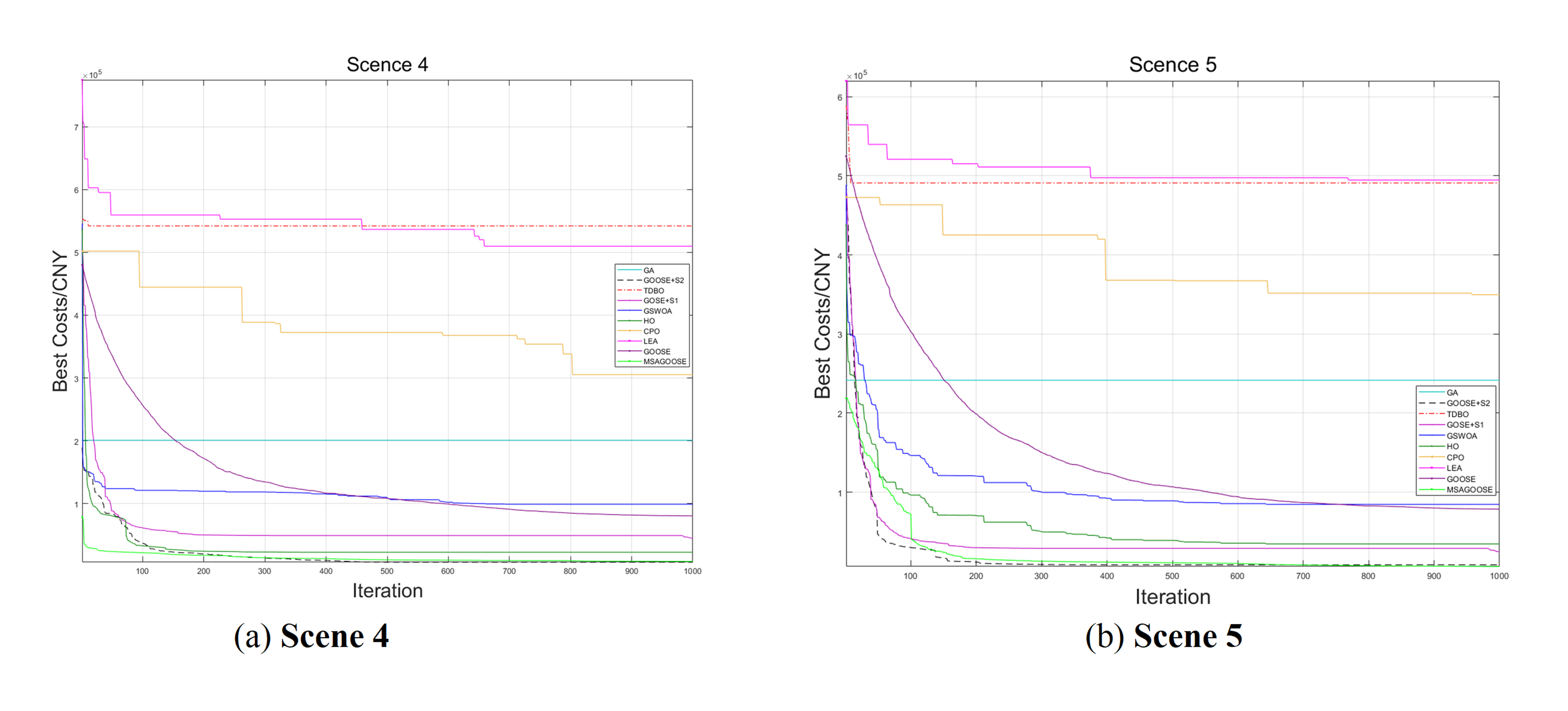

Supplement: S1 Fig — (ZIP) [file pone.0331927.s001.zip › S1 Fig/Fig6.tif]

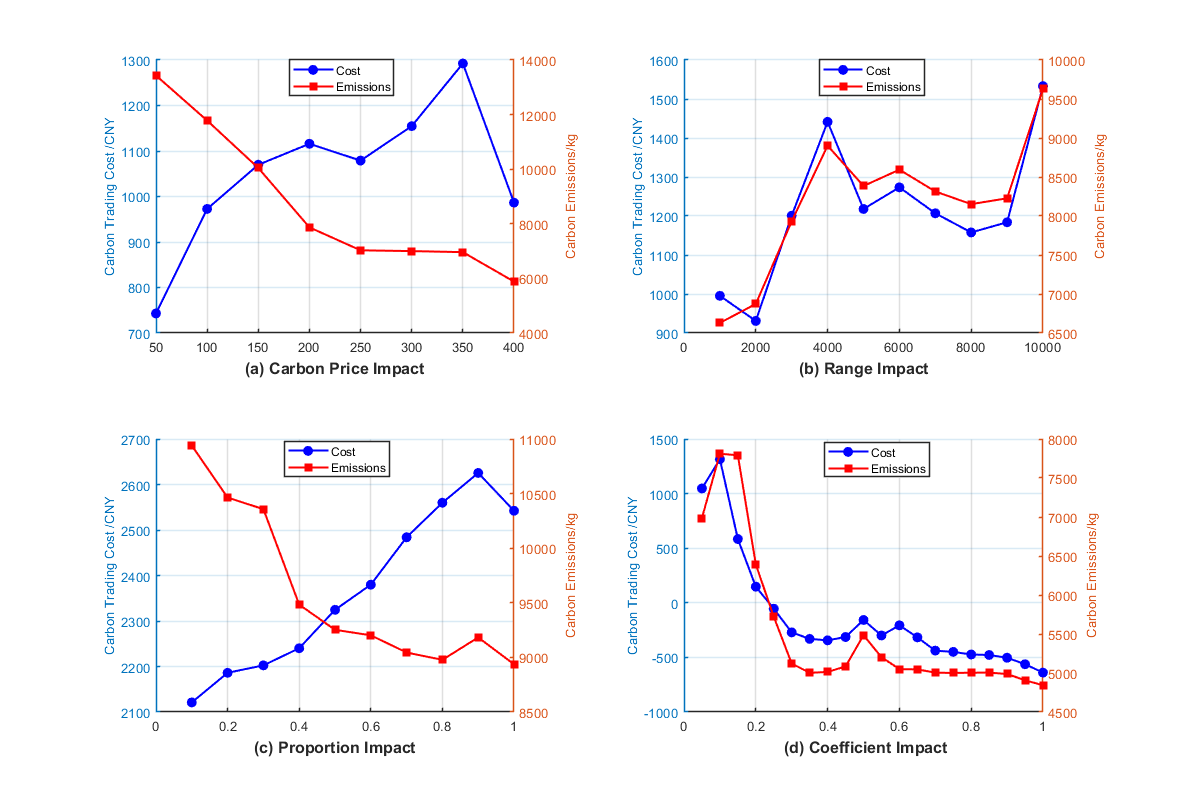

Supplement: S1 Fig — (ZIP) [file pone.0331927.s001.zip › S1 Fig/Fig7.tif]

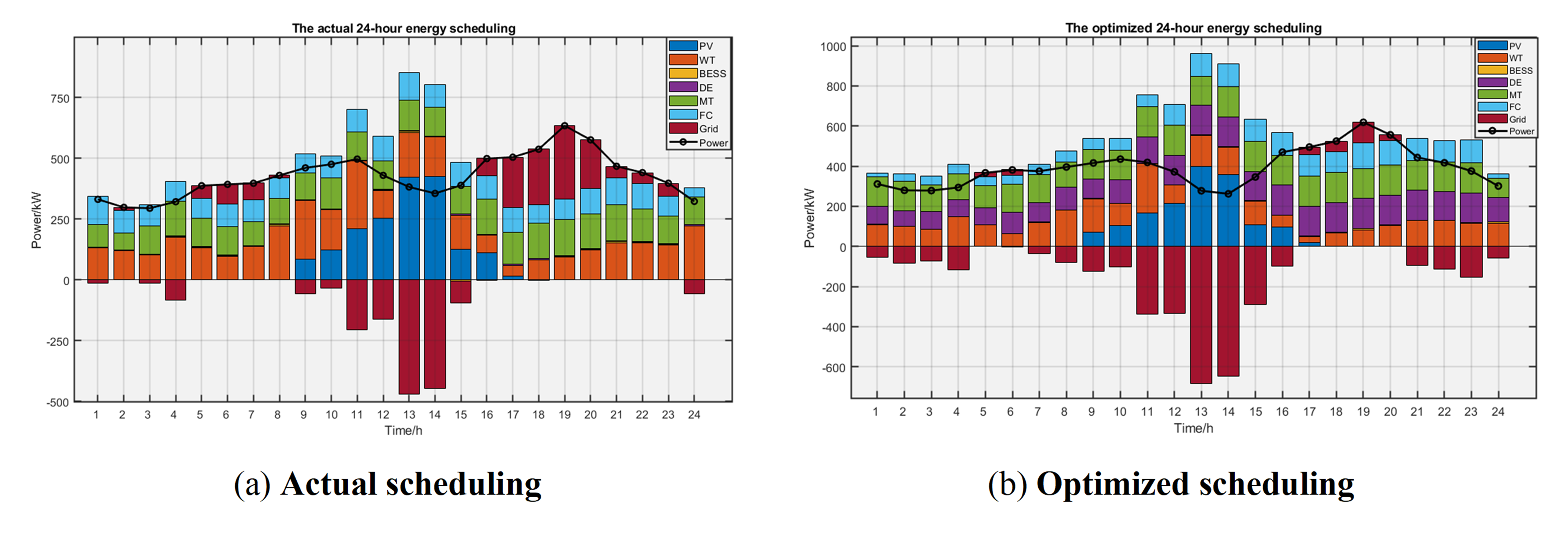

Supplement: S1 Fig — (ZIP) [file pone.0331927.s001.zip › S1 Fig/Fig8.tif]
