# Supplementary material for: Low-carbon optimal scheduling of integrated energy systems based on multi-strategy ameliorated goose algorithm and green certificate-carbon trading coordination
Source: PLoS One. 2025 Sep 12;20(9):e0331927. doi: 10.1371/journal.pone.0331927 (PMC12431214; doi:10.1371/journal.pone.0331927)
Supplement: S1 Table — (DOCX) [file pone.0331927.s002.docx]

**Table 1. The introduction of PDFs**

| **Distribution Type​​** | **Sampling Formula​​** | **Dynamic Parameter Adjustment​​** | **​​Role​​** |
| --- | --- | --- | --- |
| **Gaussian Distribution​​** | **ΔS~N(0,σt2)** | **σt=σ0*e−0.01it** | **Fine-step convergence, suppresses oscillation** |
| **Cauchy Distribution​​** | **ΔS=γ*tan(π(U−0.5))** | **γ=2−(it/T)​** | **Heavy-tailed properties, escape local optima** |
| **Pareto Distribution​​** | **Δs=Xm*(U−1/α−1)** | **α=1.5+0.5sin(πt/2π)** | **Long-distance jumps, explore unknown regions** |

**Table 2 Hyperparameters of various algorithms**

| Algorithm | Hyperparameters | Convergence Criteri | |
| --- | --- | --- | --- |
| GA | **p_c = 0.9, p_m = 0.01** | | **Loops = *Maxit* or or** *δ*< **1e-6 over 30 iterations** |
| TDBO | **P= 0.2; k = 5; w1 = 0.5; w2 =0.1** | | **Loops = *Maxit* or or** *δ*< **1e-6 over 30 iterations** |
| GSWOA | **w_max=0.2-0.5; b_base=0.75; P=0.3-0.7** | | **Loops = *Maxit* or or** *δ*< **1e-6 over 30 iterations** |
| LEA | **h_max = 0.7; h_min = 0; λ_c=0.5; λ_p=0.5** | | **Loops = *Maxit* or or** *δ*< **1e-6 over 30 iterations** |
| CPO | **T = 2；step = 0.02；α = 0.2；Tf = 0.8** | | **Loops = *Maxit* or or** *δ*< **1e-6 over 30 iterations** |
| HO | **b∈[2, 4]; c∈[1, 1.5]; d∈[2, 3]; l∈[-2π, 2π]** | | **Loops = *Maxit* or or** *δ*< **1e-6 over 30 iterations** |
| GOOSE | **coe=0.83; Weight_Stone=12; pro=0.2; rnd=0.5** | | **Loops = *Maxit* or or** *δ*< **1e-6 over 30 iterations** |
| MSAGOOSE | **coe=0.83; Weight_Stone=12; pro=0.2;rnd=p** | | **Loops = *Maxit* or or** *δ*< **1e-6 over 30 iterations** |

**Table 3. CEC2022 and CEC2017 benchmark functions**

| Category | No. | Functions | Fi* |
| --- | --- | --- | --- |
| CEC2022  (Dim=20) | **4** | **Shifted and full Rotated Non-Continuous Rastrigin’s Function** | **800** |
|  | **8** | **Hybrid Function 3 ( N = 5)** | **2200** |
|  | **12** | **Composition Function 4 (N = 6)** | **2700** |
| CEC2017  (Dim=100) | **7** | **Shifted and Rotated Lunacek Bi-Rastrigin Function** | **700** |
|  | **19** | **Hybrid Function 6 (N=5)** | **1900** |
|  | **30** | **Composition Function 10 (N=3)** | **3000** |
|  |  | **Search range: [−100,100]** |  |

**Table 4. Experimental results on CEC2022 and CEC2017**

| Functons | Agorithm | | | | | | | | | | |
| --- | --- | --- | --- | --- | --- | --- | --- | --- | --- | --- | --- |
|  |  | GA | S2 | TDBO | S1 | GSWOA | HO | CPO | LEA | GOOSE | MSAGOOSE |
| F4 | **Opt** | **898** | **841** | **899** | **856** | **907** | **839** | **916** | **855** | **891** | **800** |
|  | **Ave** | **933** | **858** | **923** | **882** | **931** | **886** | **921** | **873** | **901** | **801** |
|  | **Std** | **1.43** | **15.27** | **17.08** | **13.9** | **21.86** | **10.22** | **26.18** | **15.17** | **23.1** | **24.42** |
|  | **Fri** | **8.7** | **2.75** | **7.9** | **4.15** | **8.8** | **4.35** | **7.9** | **3.45** | **5.95** | **1.05** |
|  | **Rank** | **9** | **2** | **7** | **4** | **10** | **5** | **8** | **3** | **6** | **1** |
|  | ***p*** | **2.33E-11** | **2.33E-11** | **2.33E-11** | **2.33E-11** | **2.33E-11** | **2.33E-11** | **2.33E-11** | **2.33E-11** | **2.33E-11** |  |
| F8 | **Opt** | **2256** | **2223** | **2246** | **2227** | **2229** | **2239** | **2240** | **2249** | **2455** | **2200** |
|  | **Ave** | **2299** | **2259** | **2251** | **2229** | **2238** | **2241** | **2241** | **2278** | **2531** | **2201** |
|  | **Std** | **63.82** | **57.11** | **6.11** | **5.28** | **7.84** | **9.43** | **2.93** | **53.95** | **70.52** | **0.33** |
|  | **Fri** | **7.05** | **4.10** | **7.30** | **2.85** | **5.00** | **5.20** | **5.95** | **6.55** | **10.00** | **1.00** |
|  | **Rank** | **8** | **3** | **9** | **2** | **4** | **5** | **6** | **7** | **10** | **1** |
|  | ***p*** | **2.96E-07** | **2.96E-07** | **2.96E-07** | **2.96E-07** | **2.96E-07** | **2.96E-07** | **2.96E-07** | **2.96E-07** | **2.96E-07** |  |
| F12 | **Opt** | **2914** | **2801** | **2795** | **2836** | **2947** | **2837** | **3105** | **2892** | **4601** | **2700** |
|  | **Ave** | **3158** | **2890** | **2884** | **2889** | **3533** | **2899** | **2962** | **2861** | **5468** | **2732** |
|  | **Std** | **72.12** | **113.36** | **32.57** | **44.20** | **220.85** | **42.64** | **12.12** | **21.01** | **387.59** | **9.65** |
|  | **Fri** | **8.00** | **3.50** | **4.20** | **4.30** | **9.00** | **4.60** | **6.55** | **3.15** | **10.00** | **1.70** |
|  | **Rank** | **8** | **3** | **4** | **5** | **9** | **6** | **7** | **2** | **10** | **1** |
|  | ***p*** | **3.02E-11** | **3.82E-09** | **3.02E-11** | **3.82E-09** | **3.02E-11** | **3.02E-11** | **3.02E-11** | **3.02E-11** | **3.02E-11** |  |
| F7 | **Opt** | **2250** | **739** | **2460** | **1486** | **3012** | **2579** | **1778** | **2317** | **2392** | **703** |
|  | **Ave** | **2436** | **799** | **2533** | **1613** | **3055** | **2632** | **1812** | **2431** | **2449** | **715** |
|  | **Std** | **370.73** | **80.28** | **146.92** | **253.24** | **86.19** | **105.38** | **67.88** | **227.07** | **112.70** | **76.23** |
|  | **Fri** | **6.57** | **1.87** | **7.10** | **3.23** | **9.90** | **8.13** | **3.77** | **6.73** | **6.57** | **1.17** |
|  | **Rank** | **5** | **2** | **8** | **3** | **10** | **9** | **4** | **7** | **6** | **1** |
|  | ***p*** | **3.02E-11** | **3.02E-11** | **3.02E-11** | **3.02E-11** | **3.02E-11** | **3.02E-11** | **3.02E-11** | **3.02E-11** | **3.02E-11** |  |
| F19 | **Opt** | **2049** | **2009** | **2001** | **1979** | **1988** | **1991** | **1991** | **2028** | **2281** | **1951** |
|  | **Ave** | **8.08E+06** | **3.85E+03** | **9.08E+08** | **1.82E+06** | **2.08E+10** | **1.63E+07** | **3.56E+07** | **1.28E+07** | **8.04E+05** | **2.00E+03** |
|  | **Std** | **1.23E+06** | **2.43E+03** | **2.45E+07** | **1.69E+05** | **2.08E+08** | **9.94E+05** | **1.14E+06** | **6.01E+05** | **1.08E+05** | **1.04E+03** |
|  | **Fri** | **5.33** | **1.83** | **9.00** | **3.93** | **10.00** | **6.43** | **7.77** | **6.27** | **3.27** | **1.17** |
|  | **Rank** | **5** | **2** | **9** | **4** | **10** | **7** | **8** | **6** | **3** | **1** |
|  | ***p*** | **3.02E-11** | **1.86E-10** | **3.02E-11** | **1.86E-10** | **7.69E-11** | **3.02E-11** | **3.02E-11** | **3.02E-11** | **3.02E-11** |  |
| F30 | **Opt** | **6.38E+06** | **5.09E+03** | **8.60E+08** | **7.23E+05** | **1.69E+10** | **1.24E+08** | **1.96E+08** | **1.84E+07** | **2.02E+06** | **5.09E+03** |
|  | **Ave** | **4.90E+07** | **1.01E+04** | **2.32E+09** | **2.09E+07** | **2.36E+10** | **2.69E+08** | **3.35E+08** | **7.23E+07** | **2.91E+06** | **9.98E+03** |
|  | **Std** | **3.08E+07** | **5.86E+03** | **7.53E+08** | **2.35E+07** | **5.26E+09** | **9.75E+07** | **8.60E+07** | **2.96E+07** | **4.64E+05** | **5.43E+03** |
|  | **Fri** | **5.10** | **1.90** | **9.00** | **4.13** | **10.00** | **7.23** | **7.77** | **5.63** | **3.13** | **1.10** |
|  | **Rank** | **5** | **2** | **9** | **4** | **10** | **7** | **8** | **6** | **3** | **1** |
|  | ***p*** | **1.88E-11** | **5.83E-10** | **2.56E-11** | **5.83E-10** | **1.88E-11** | **2.41E-11** | **2.51E-10** | **1.66E-11** | **1.88E-10** |  |

**Table 5. Time-of-use electricity price**

| **Period** | **Time** | **Purchase (CNY/kWh)** | **Sell (CNY/kWh)** |
| --- | --- | --- | --- |
| **Valley Period** | **0:00-7:00;23:00-24:00** | **0.52** | **0.39** |
| **Normal Period** | **7:00-10:00;15:00-18:00;21:00-23:00** | **0.94** | **0.51** |
| **Peak Period** | **10:00-15:00;18:00-21:00** | **1.24** | **1.06** |
|  |  |  |  |

**Table 6. Pollutant treatment cost and emission coefficient [43]**

| **Pollutant** | **Pollutant treatment costs** | **Discharge ratio(g/kwh)** | | | |
| --- | --- | --- | --- | --- | --- |
|  |  | **MT** | **FC** | **Grid** | **DE** |
| **CO** | **10.17** | **0.049** | **0** | **0.082** | **435.2** |
| **NOₓ** | **13.213** | **0.29** | **0.019** | **1.47** | **10.09** |
| **SO₂** | **67.138** | **0.0039** | **0.0035** | **1.34** | **0.306** |

**Table 7. Related operating parameters of power supply equipment**

| Type | DE | MT | FC | Grid |
| --- | --- | --- | --- | --- |
| Lower power limit/kW | **3** | **3** | **3** | **-300** |
| Upper power limit/kW | **150** | **150** | **150** | **300** |
| Limit of climbing power/(kW/min) | **1.5** | **1.5** | **1.5** |  |
| Operation coefficient (CNY/kW) | **0.128** | **0.0489** | **0.0288** |  |

**Table 8. Energy storage parameters**

| Type | Parameter | Value | Parameter | Value |
| --- | --- | --- | --- | --- |
| BESS | **Soc_max_/ (kW·h)** | **150** | **P_BESS, min_ / kW** | **-150** |
|  | **Soc_min_ / (kW·h)** | **0** | **Efficiency** | **0.9** |
|  | **P_BESS, max_ / kW** | **150** |  |  |

**Table 9. Scene setting information**

| **Scene** | **Operating cost** | **Environment Penalty** | **CET** | **GCT** | **Improved CET CostCost** |
| --- | --- | --- | --- | --- | --- |
| **Scene 1** | **✓** |  |  |  |  |
| **Scene 2** | **✓** | **✓** |  |  |  |
| **Scene 3** | **✓** | **✓** | **✓** |  |  |
| **Scene 4** | **✓** | **✓** | **✓** | **✓** |  |
| **Scene 5** | **✓** | **✓** |  | **✓** | **✓** |

**Table 10. Comparison of the convergence curves between the algorithms**

|  |  | **GA** | **GOOSE +S2** | **TDBO** | **GOOSE +S1** | **GSWOA** | **HO** | **CPO** | **LEA** | **GOOSE** | **MSAGOOSE** |
| --- | --- | --- | --- | --- | --- | --- | --- | --- | --- | --- | --- |
| **Scene 4** | **Opt/CNY** | **2.05E+05** | **9.99E+03** | **5.47E+04** | **4.45E+04** | **9.89E+04** | **2.25E+04** | **3.09E+05** | **5.20E+05** | **8.05E+04** | **7.94E+03** |
|  | **time/s** | **153.23** | **152.11** | **169.45** | **168.4** | **167.4** | **171.4** | **165.34** | **163.24** | **154.8** | **155.33** |
| **Scene 5** | **Opt/CNY** | **2.42E+05** | **8.26E+03** | **4.91E+04** | **2.44E+04** | **8.46E+04** | **3.46E+04** | **3.43E+05** | **4.97E+05** | **7.86E+04** | **6.15E+03** |
|  | **time/s** | **153.65** | **153.15** | **165.72** | **166.4** | **164.9** | **164.4** | **163.83** | **162.78** | **155** | **155.73** |

**Table 11. Comparison of multi-scene operation results**

| **Scene** | **1** | **2** | **3** | **4** | **5** |
| --- | --- | --- | --- | --- | --- |
| **Carbon emissions/kg** | **18894.91** | **11634.36** | **5887.19** | **5840.89** | **5121.34** |
| **SO2/kg** | **3.44** | **2.92** | **3.77** | **3.35** | **2.60** |
| **NOX/kg** | **33.80** | **4.88** | **6.42** | **5.94** | **4.95** |
| **CO/kg** | **1298.60** | **35.43** | **64.32** | **63.52** | **32.82** |
| **Green power rate** | **0.79** | **0.77** | **0.74** | **0.83** | **0.88** |
| **Operating cost/CNY** | **8183.37** | **7892.06** | **7332.80**  **considering integrated demand**  **response 7332.80** | **7294.49** | **6658.94** |
| **Carbon trading cost/CNY** |  |  | **341.31** | **466.94** | **-270.67** |
| **Green certificate cost/CNY** |  |  |  | **-770.86** | **-841.85** |
| **Environment Penalty /CNY** |  | **620.90** | **1003.54** | **946.95** | **604.80** |
| **Total cost/CNY** | **8183.37** | **8512.96** | **8677.65** | **7937.51** | **6151.23** |
