# Supplementary material for: Low-carbon optimal scheduling of integrated energy systems based on multi-strategy ameliorated goose algorithm and green certificate-carbon trading coordination
Source: PLoS One. 2025 Sep 12;20(9):e0331927. doi: 10.1371/journal.pone.0331927 (PMC12431214; doi:10.1371/journal.pone.0331927)
Supplement: Attachment — Submitted filename: Response to Reviewers.docx [file pone.0331927.s005.docx]

Contents

[Reviewer #1	2](#_Toc205928578)

[Reviewer #2	8](#_Toc205928579)

**Manuscript：*Low-carbon optimal scheduling of integrated energy systems based on multi-strategy ameliorated goose algorithm and green certificate-carbon trading coordination***

**Manuscript ID：PONE-D-25-37059**

Dear Editor and Reviewers,

We gratefully appreciate the editor and all reviewers for their time spent making positive and constructive comments. These comments are all valuable and helpful for revising and improving our manuscript entitled “***Low-carbon optimal scheduling of integrated energy systems based on multi-strategy ameliorated goose algorithm and green certificate-carbon trading coordination***” (ID: PONE-D-25-37059) for publication in ***PLOS ONE***, as well as the important guiding significance to our research. We have carefully considered the feedback from the reviewers and have made substantial revisions to enhance the clarity, coherence, and scientific rigor of our work.

The revised sections are highlighted in yellow. Attached please find the revised version, which we would like to submit for your kind consideration.

Thank you once again for the opportunity to improve our manuscript. We look forward to the possibility of our revised work being published in ***PLOS ONE***.

Best regards.

Sincerely,

Chengcheng Ding

Yun ZHU

-------------------------------------------------------------------------------------------------------

The following is a point-to-point response to reviewers’ comments.

**Response to reviewers:**

# Reviewer #1:

1. The abstract is overly lengthy and, although information-dense, lacks clear logical organization. It is recommended to adopt a four-part structure — Background, Method, Results, and Conclusion — to present the content more clearly.

Authors’ Answer: Thank you very much to the expert for your valuable comments.

The author has thoroughly rewritten the abstract using the recommended four-part structure. The revised abstract now concisely covers the background, methodology, main results, and conclusions, thereby improving readability and logical flow. The author has rewritten the abstract as follows (Page 1, Line 10 to Line 26):

Background:

Integrated energy systems (IES) in China face a dual challenge under the “dual-carbon” targets: maximizing renewable energy utilization while minimizing carbon emissions. Traditional tiered carbon markets often lack the flexibility to dynamically incentivize low-carbon operation.

Method:

To address this, a coordinated framework is proposed, integrating a dynamic carbon emission trading (CET) mechanism with green certificate trading (GCT) and a Multi-Strategy Ameliorated Goose Optimization (MSAGOOSE) algorithm. The GCT-CET mechanism introduces exponential reward–penalty coefficients based on real-time renewable consumption rates, enabling adaptive carbon pricing. MSAGOOSE combines adaptive parameter adjustment, multimodal distribution-guided exploration, and population-aware reverse learning to improve optimization robustness in high-dimensional, nonlinear scheduling problems.

Results:

Benchmark evaluations on CEC2017 and CEC2022 show that MSAGOOSE achieves an order-of-magnitude improvement in accuracy over seven state-of-the-art algorithms. In a 24-hour IES scheduling case in Anhui Province, the proposed method reduces carbon emissions by 27.3% (5,121 kg/d), increases renewable energy share to 88%, and cuts operating costs by 24.8% (6,151 CNY/d). Parametric analysis further confirms the framework’s effectiveness in balancing economic and environmental goals under decentralized energy scenarios.

Conclusion:

This study presents a policy-algorithm co-design paradigm that offers both theoretical and practical support for low-carbon IES transitions, enabling scalable, flexible, and economically viable scheduling strategies.

2. It is recommended that the authors enrich the Introduction by incorporating recent studies on multi-agent game-theoretic approaches—such as asymmetric Nash bargaining, and Cournot–Nash competition—in the context of integrated energy systems. This would broaden the modeling perspective and provide a solid theoretical foundation for future research directions.
[1] A Distributed Market-Aided Restoration Approach of Multi-Energy Distribution Systems Considering Comprehensive Uncertainties from Typhoon Disaster.

[2] Risk-averse stochastic capacity planning and P2P trading collaborative optimization for multi-energy microgrids considering carbon emission limitations: An asymmetric Nash bargaining approach.

Authors’ Answer: Thank you very much to the expert for your valuable comments.The literatures proposed by the experts are very forward-looking and creative. The author quotes these documents and highly praises the contribution of these documents to the development of the current energy industry. The details are as follows:

(1) Reference [6,7], based on a non-Nash equilibrium, incorporates electricity-heat bidding within a distributed market-assisted recovery framework for multi-energy systems, providing a creative template for IES scheduling. (Page 2, line 43 to line 45)

[6] Wang Z, Hou H, Wei R, et al. A Distributed Market-Aided Restoration Approach of Multi-Energy Distribution Systems Considering Comprehensive Uncertainties from Typhoon Disaster[J]. IEEE Transactions on Smart Grid, 2025.

[7] Hou H, Wang Z, Zhao B, et al. Peer-to-peer energy trading among multiple microgrids considering risks over uncertainty and distribution network reconfiguration: A fully distributed optimization method[J]. International Journal of Electrical Power & Energy Systems, 2023, 153: 109316.

(2) The research suggests using asymmetric Nash bargaining to distribute benefits fairly in carbon market transactions, and its "contribution-bargaining ability" mapping technique may help integrate dynamic carbon markets (Page 2, line 63 to line 66)

[26] Wang Z, Hou H, Zhao B, et al. Risk-averse stochastic capacity planning and P2P trading collaborative optimization for multi-energy microgrids considering carbon emission limitations: An asymmetric Nash bargaining approach[J]. Applied Energy, 2024, 357: 122505.

3. Although the optimization model includes power balance and ramping constraints, it omits essential practical constraints such as SOC limits for storage and dynamic bounds for generation. Please enrich the model description to enhance engineering fidelity.

Authors’ Answer: Thank you very much to the expert for your valuable comments. In order to enhance the fidelity and reproducibility of the research, the author enriches the description of relevant constraints and supplements the constraint function model in section 2.2. In section 5, the related parameters, such as the stored SOC limit and the dynamic limit of power generation, are supplemented. Details are as follows:

1) Model supplement (Page 7, line 215 to line 221):

(2) Power output constraint

$$\left\{\begin{matrix} P_{i,t}^{\min}\leq P_{i,t}\leq P_{i,t}^{\max} \\ |P_{i,t}-P_{i,t-1}|\leq R_{i}\Delta t \end{matrix}\right.$$

In the formula, $P_{i,t}^{\min}$ and $P_{i}^{\max}$ represent the upper and lower limits of each power output.$R_{i}$ represents the limits of each power output.

(3) Energy storage operation constraint

In the formula, $P_{bess}^{\text{min}}$ and $P_{bess}^{\text{max}}$ are the minimum and maximum output power of the energy storage unit, respectively; $Soc^{\text{min}}$and $Soc^{\text{max}}$are the lower and upper limits of the capacity of the energy storage unit, respectively.

2) Data imputation (Page 16-17, line 399 to line 401)

Table 7. Related operating parameters of power supply equipment

| Type | DE | MT | FC | Grid |
| --- | --- | --- | --- | --- |
| Lower power limit/kW | 3 | 3 | 3 | -300 |
| Upper power limit/kW | 150 | 150 | 150 | 300 |
| Limit of climbing power/(kW/min) | 1.5 | 1.5 | 1.5 |  |
| Operation coefficient (CNY/kW) | 0.128 | 0.0489 | 0.0288 |  |

Table 8. Energy storage parameters

| Type | Parameter | Value | Parameter | Value |
| --- | --- | --- | --- | --- |
| BESS | Socmax/ (kW·h) | 150 | PBESS, min / kW | -150 |
|  | Socmin / (kW·h) | 0 | Efficiency | 0.9 |
|  | PBESS, max / kW | 150 |  |  |

4. The manuscript lacks a unified modeling framework diagram. It is recommended to include a schematic diagram that connects the incentive mechanism, scheduling model, and optimization algorithm to highlight the closed-loop structure of policy response and optimization.

Authors’ Answer: Thank you very much for your valuable and constructive comments. The author combs through the whole article in detail, and expands the flow chart of the original algorithm, connects the schematic diagram of the incentive mechanism, scheduling model, and optimization algorithm, and constructs a closed-loop structure that highlights policy response and optimization. The final results are as follows (Page 11, line 320):

**Fig 3.** **The flow chart of the IES program solved by the MSAGOOSE algorithm**

5. Algorithm performance testing should clarify how hyperparameters were tuned, how convergence was judged, and whether statistical significance was verified across runs.

Authors’ Answer: Thank you very much for your valuable and constructive comments. For hyperparameter adjustment, the author joined the relevant introduction. For the problem of convergence judgment, the author supplements the formula of convergence judgment and its introduction. In order to verify the statistical significance, the author added the *p* and the Wilcoxon rank-sum test experimental data into Table 4, and verified the superiority and reliability of the improved algorithm to ensure the reproducibility of the results. The statistical significance was verified. The specific results are as follows：

1) Hyperparameter description

The selection of hyperparameters is guided by the literature and CEC test function, and screened by the grid experiment method to ensure its reliability and portability in different scenarios. (Page 12, line 342 to line 344)

**Table 2 Hyperparameters of various algorithms** (Page 12-13, line 348)

| Algorithm | Hyperparameters | Convergence Criteria | |
| --- | --- | --- | --- |
| GA | **p_c = 0.9, p_m = 0.01** | | **Loops = *Maxit* or or** *δ*< **1e-6 over 30 iterations** |
| TDBO | **P= 0.2; k = 5; w1 = 0.5; w2 =0.1** | | **Loops = *Maxit* or or** *δ*< **1e-6 over 30 iterations** |
| GSWOA | **w_max∈[0.2, 0.5]; b_base=0.75; P=∈[0.3, 0.7]** | | **Loops = *Maxit* or or** *δ*< **1e-6 over 30 iterations** |
| LEA | **h_max = 0.7; h_min = 0; λ_c =0.5; λ_p=0.5** | | **Loops = *Maxit* or or** *δ*< **1e-6 over 30 iterations** |
| CPO | **T = 2；step = 0.02；α = 0.2；Tf = 0.8** | | **Loops = *Maxit* or or** *δ*< **1e-6 over 30 iterations** |
| HO | **b∈[2, 4]; c∈[1, 1.5]; d∈[2, 3]; l∈[-2π, 2π]** | | **Loops = *Maxit* or or** *δ*< **1e-6 over 30 iterations** |
| GOOSE | **coe=0.83; Weight_Stone=12; pro=0.2; rnd=0.5** | | **Loops = *Maxit* or or** *δ*< **1e-6 over 30 iterations** |
| MSAGOOSE | **coe=0.83; Weight_Stone=12; pro=0.2; rnd=p** | | **Loops = *Maxit* or or** *δ*< **1e-6 over 30 iterations** |

2) Judge convergence (Page 8, line 249 to line 253):

The convergence condition of the algorithm is：

When the relative change rate of the target value converges or the number of cycles is greater than $MaxIt$the cycle ends and is judged to be convergent. This is one of the most commonly used criteria for iterative algorithms.

3) Verify statistical significance (Page 14, line 354 to line 357):

Table 4. Experimental results on CEC2022 and CEC2017 (Page 13-14, line 352)

| Functons | Agorithm | | | | | | | | | | |
| --- | --- | --- | --- | --- | --- | --- | --- | --- | --- | --- | --- |
|  |  | GA | S2 | TDBO | S1 | GSWOA | HO | CPO | LEA | GOOSE | MSAGOOSE |
| F4 | Opt | 898 | 841 | 899 | 856 | 907 | 839 | 916 | 855 | 891 | 800 |
|  | Ave | 933 | 858 | 923 | 882 | 931 | 886 | 921 | 873 | 901 | 801 |
|  | Std | 1.43 | 15.27 | 17.08 | 13.9 | 21.86 | 10.22 | 26.18 | 15.17 | 23.1 | 24.42 |
|  | Fri | 8.7 | 2.75 | 7.9 | 4.15 | 8.8 | 4.35 | 7.9 | 3.45 | 5.95 | 1.05 |
|  | Rank | 9 | 2 | 7 | 4 | 10 | 5 | 8 | 3 | 6 | 1 |
|  | *p* | 2.33E-11 | 2.33E-11 | 2.33E-11 | 2.33E-11 | 2.33E-11 | 2.33E-11 | 2.33E-11 | 2.33E-11 | 2.33E-11 |  |
| F8 | Opt | 2256 | 2223 | 2246 | 2227 | 2229 | 2239 | 2240 | 2249 | 2455 | 2200 |
|  | Ave | 2299 | 2259 | 2251 | 2229 | 2238 | 2241 | 2241 | 2278 | 2531 | 2201 |
|  | Std | 63.82 | 57.11 | 6.11 | 5.28 | 7.84 | 9.43 | 2.93 | 53.95 | 70.52 | 0.33 |
|  | Fri | 7.05 | 4.10 | 7.30 | 2.85 | 5.00 | 5.20 | 5.95 | 6.55 | 10.00 | 1.00 |
|  | Rank | 8 | 3 | 9 | 2 | 4 | 5 | 6 | 7 | 10 | 1 |
|  | *p* | 2.96E-07 | 2.96E-07 | 2.96E-07 | 2.96E-07 | 2.96E-07 | 2.96E-07 | 2.96E-07 | 2.96E-07 | 2.96E-07 |  |
| F12 | Opt | 2914 | 2801 | 2795 | 2836 | 2947 | 2837 | 3105 | 2892 | 4601 | 2700 |
|  | Ave | 3158 | 2890 | 2884 | 2889 | 3533 | 2899 | 2962 | 2861 | 5468 | 2732 |
|  | Std | 72.12 | 113.36 | 32.57 | 44.20 | 220.85 | 42.64 | 12.12 | 21.01 | 387.59 | 9.65 |
|  | Fri | 8.00 | 3.50 | 4.20 | 4.30 | 9.00 | 4.60 | 6.55 | 3.15 | 10.00 | 1.70 |
|  | Rank | 8 | 3 | 4 | 5 | 9 | 6 | 7 | 2 | 10 | 1 |
|  | *p* | 3.02E-11 | 3.82E-09 | 3.02E-11 | 3.82E-09 | 3.02E-11 | 3.02E-11 | 3.02E-11 | 3.02E-11 | 3.02E-11 |  |
| F7 | Opt | 2250 | 739 | 2460 | 1486 | 3012 | 2579 | 1778 | 2317 | 2392 | 703 |
|  | Ave | 2436 | 799 | 2533 | 1613 | 3055 | 2632 | 1812 | 2431 | 2449 | 715 |
|  | Std | 370.73 | 80.28 | 146.92 | 253.24 | 86.19 | 105.38 | 67.88 | 227.07 | 112.70 | 76.23 |
|  | Fri | 6.57 | 1.87 | 7.10 | 3.23 | 9.90 | 8.13 | 3.77 | 6.73 | 6.57 | 1.17 |
|  | Rank | 5 | 2 | 8 | 3 | 10 | 9 | 4 | 7 | 6 | 1 |
|  | *p* | 3.02E-11 | 3.02E-11 | 3.02E-11 | 3.02E-11 | 3.02E-11 | 3.02E-11 | 3.02E-11 | 3.02E-11 | 3.02E-11 |  |
| F19 | Opt | 2049 | 2009 | 2001 | 1979 | 1988 | 1991 | 1991 | 2028 | 2281 | 1951 |
|  | Ave | 8.08E+06 | 3.85E+03 | 9.08E+08 | 1.82E+06 | 2.08E+10 | 1.63E+07 | 3.56E+07 | 1.28E+07 | 8.04E+05 | 2.00E+03 |
|  | Std | 1.23E+06 | 2.43E+03 | 2.45E+07 | 1.69E+05 | 2.08E+08 | 9.94E+05 | 1.14E+06 | 6.01E+05 | 1.08E+05 | 1.04E+03 |
|  | Fri | 5.33 | 1.83 | 9.00 | 3.93 | 10.00 | 6.43 | 7.77 | 6.27 | 3.27 | 1.17 |
|  | Rank | 5 | 2 | 9 | 4 | 10 | 7 | 8 | 6 | 3 | 1 |
|  | *p* | 3.02E-11 | 1.86E-10 | 3.02E-11 | 1.86E-10 | 7.69E-11 | 3.02E-11 | 3.02E-11 | 3.02E-11 | 3.02E-11 |  |
| F30 | Opt | 6.38E+06 | 5.09E+03 | 8.60E+08 | 7.23E+05 | 1.69E+10 | 1.24E+08 | 1.96E+08 | 1.84E+07 | 2.02E+06 | 5.09E+03 |
|  | Ave | 4.90E+07 | 1.01E+04 | 2.32E+09 | 2.09E+07 | 2.36E+10 | 2.69E+08 | 3.35E+08 | 7.23E+07 | 2.91E+06 | 9.98E+03 |
|  | Std | 3.08E+07 | 5.86E+03 | 7.53E+08 | 2.35E+07 | 5.26E+09 | 9.75E+07 | 8.60E+07 | 2.96E+07 | 4.64E+05 | 5.43E+03 |
|  | Fri | 5.10 | 1.90 | 9.00 | 4.13 | 10.00 | 7.23 | 7.77 | 5.63 | 3.13 | 1.10 |
|  | Rank | 5 | 2 | 9 | 4 | 10 | 7 | 8 | 6 | 3 | 1 |
|  | *p* | 1.88E-11 | 5.83E-10 | 2.56E-11 | 5.83E-10 | 1.88E-11 | 2.41E-11 | 2.51E-10 | 1.66E-11 | 1.88E-10 |  |

The *p* denotes the Wilcoxon rank-sum test, a non-parametric check; when p < 0.05, we reject the null hypothesis and deem the improvement statistically significant rather than random. All *p*-values are far below 1 × 10⁻⁶, confirming MSAGOOSE’s superiority is highly significant across repeated runs.

# Reviewer #2:

1. Although the introduction cites many references, few of them directly support the research motivation or identified limitations. It is recommended to streamline general references and supplement targeted literature on scheduling mechanisms, carbon market integration, and algorithmic performance issues.

Authors’ Answer: Thank you very much for your valuable and constructive comments. In response to this problem, the author re-combed the content of the introduction, and re-analyzed the references cited in this paragraph according to the core of each paragraph, and deleted the original seven documents with weak support and honor. Among them, the references [1],[4], and [5] mainly provide macro policy and technical trend background, but it is not directly related to the core GCT-CET mechanism construction, IES scheduling modeling, and algorithm improvement. Although it is helpful to introduce the background, too many references in the introduction will scatter the pertinence of the research motivation, so it is streamlined. references [6] are also redundancy problems; in order to simplify the literature, delete. References [20], [21] and [24] mainly introduce the algorithm, which are not related to the IES in this paper, and are also deleted.

At the same time, in order to strengthen the support of research motivation, on the basis of streamlining the literature, the author re-read and collected targeted literature on scheduling mechanisms, carbon market integration and algorithm performance issues. After careful screening, some relatively good literatures were selected to support this study.Then, we include

(1) references [6], [7], [9], [10], [12] and [13] on the scheduling mechanism of integrated energy system (IES), including the existing mechanism innovation and the problem of its scheduling mechanism, which directly connects the research direction of the scheduling mechanism in this paper.
(2) references [22], [23], [25] and [26] directly link the carbon market mechanism with scheduling, including dynamic GCT-CET interaction and market optimization under emission constraints, pointing out the rigidness of the current two mechanisms and the narrowness of the linkage mechanism.
(3) The latest progress in the performance of the swarm intelligence algorithm, GOOSE original formula, and the multi-strategy improvement of high-dimensional optimization in References [27] - [32]. These additions directly strengthen the theoretical and methodological foundations of our proposed models and algorithms, ensuring that the cited work now provides accurate support for research motivation, mechanism design, and performance verification.

The specific results are as follows：

1) Redundant literature deletion

[1] J. Peng, J. Wang, J. Qi, and Y. Liao, “Multi - stage expansion planning of distribution networks under the ‘dual - carbon’ goal,” Power System Protection and Control, vol. 50, no. 7, pp. 153–161, 2022, doi: 10.19783/j.cnki.pspc.210570.

[4] Z. Li, W. Zhang, R. Zhang, and H. Sun, “Development of renewable energy multi - energy complementary hydrogen energy system (a case study in China): a review,” Energy Exploration & Exploitation, vol. 38, no. 6, pp. 2099–2127, Nov. 2020, doi: 10.1177/0144598720953512.

[5] S. Chen, L. Xu, and H. Wu, “Theoretical interpretation and realization path of green and low - carbon transformation of China’s industrial chain under the ‘dual - carbon’ goal,” Guangdong Social Sciences, no. 5, pp. 63–74, 2024.

[6] W. Wan, L. Zhou, L. Xie, H. Bao, and Q. Zhang, “Analysis of day - ahead optimal scheduling strategy for wind - solar - diesel - storage microgrid energy management,” Solar Energy, no. 10, pp. 70–76, 2024, doi: 10.19911/j.1003 - 0417.tyn20231115.01.

[20] W. Zhang, Y. Li, K. Wang, W. Xu, and L. Gao, “A green and efficient disassembly line balancing with human-robot collaboration and destructive disassembly,” Robot. Comput-Integr. Manuf., vol. 97, p. 103081, Feb. 2026, doi: 10.1016/j.rcim.2025.103081.

[21] Wang, Q. Guo, Z. Zhou, H. Li, and D. Ma, “Optimisation investigation on hybrid battery thermal management system for electric aircraft based on genetic algorithm,” Eng. Appl. Comp. Fluid Mech., vol. 19, no. 1, p. 2517314, Dec. 2025, doi: 10.1080/19942060.2025.2517314.

[24] J. Xue and B. Shen, “Dung beetle optimizer: a new meta - heuristic algorithm for global optimization,” Journal of Supercomputing, vol. 79, no. 7, pp. 7305–7336, May 2023, doi: 10.1007/s11227 - 022 - 04959 - 6.

2) Support literature supplement

[3] Wesonga R, Tutesigensi A, Moodley K. Advancing net zero carbon construction: A techno-economic and environmental analysis of onsite microgrids and prosumer energy adoption[J]. Applied Energy, 2025, 398: 126453.

[6] Wang Z, Hou H, Wei R, et al. A Distributed Market-Aided Restoration Approach of Multi-Energy Distribution Systems Considering Comprehensive Uncertainties from Typhoon Disaster[J]. IEEE Transactions on Smart Grid, 2025.

[7] Hou H, Wang Z, Zhao B, et al. Peer-to-peer energy trading among multiple microgrids considering risks over uncertainty and distribution network reconfiguration: A fully distributed optimization method[J]. International Journal of Electrical Power & Energy Systems, 2023, 153: 109316.

[9] Yu S, Zhou S, Chen N. Multi-objective optimization of capacity and technology selection for provincial energy storage in China: The effects of peak-shifting and valley-filling[J]. Applied Energy, 2024, 355: 122289. 8

[10] Song Z, Guan X, Cheng M. Multi-objective optimization strategy for home energy management system including PV and battery energy storage[J]. Energy Reports, 2022, 8: 5396-5411 9

[12] Lou S, Yang Y, Liang Y, et al. Coordinated optimization of multi-energy generation systems considering the health damages of coal-fired pollutant dispersion[J]. CSEE Journal of Power and Energy Systems, 2021, 8(6): 1613-1622.

[13] Wang H, Xu Y, Yi Z, et al. A review on economic dispatch of power system considering atmospheric pollutant emissions[J]. Energies, 2024, 17(8): 1878

[22] Zhang W, Ji C, Liu Y, et al. Dynamic interactions of carbon trading, green certificate trading, and electricity markets: Insights from system dynamics modeling[J]. PLoS One, 2024, 19(6): e0304478.

[23] Li X, Deng J, Liu J. Energy–carbon–green certificates management strategy for integrated energy system using carbon–green certificates double-direction interaction[J]. Renewable Energy, 2025, 238: 121937.

[25] Yue X, Wang C, Sun B, et al. Synergistic effects of carbon cap-and-trade and renewable portfolio standards on renewable energy diffusion[J]. Journal of Cleaner Production, 2023, 423: 138717.

[26] Wang Z, Hou H, Zhao B, et al. Risk-averse stochastic capacity planning and P2P trading collaborative optimization for multi-energy microgrids considering carbon emission limitations: An asymmetric Nash bargaining approach[J]. Applied Energy, 2024, 357: 122505

[27] Warnakulasooriya K, Segev A. Comparative analysis of accuracy and computational complexity across 21 swarm intelligence algorithms[J]. Evolutionary Intelligence, 2025, 18(1): 18.

[29] R. K. Hamad and T. A. Rashid, “GOOSE algorithm: a powerful optimization tool for real - world engineering challenges and beyond,” Evolutionary Systems, vol. 15, no. 4, pp. 1249–1274, Aug. 2024, doi: 10.1007/s12530 - 023 - 09553 - 6.

[30] Li K. A survey of multi-objective evolutionary algorithm based on decomposition: Past and future[J]. IEEE Transactions on Evolutionary Computation, 2024.

[31] Tawhid M.A, Ibrahim A.M. An efficient hybrid swarm intelligence optimization algorithm for solving nonlinear systems and clustering problems[J]. Soft Computing, 2023, 27(13): 8867-8895.

[32] Tran D, Xie B, Zhang M. Adaptive multi-subswam optimisation for feature selection on high-dimensional classification[C]//Proceedings of the genetic and evolutionary computation conference. 2019: 481-489.

2. Section 2.2 lacks detailed analysis and structural elaboration of the proposed GCT-CET interaction mechanism. The mechanism diagram is directly adapted from prior literature, and the described improvements are vague.

Authors’ Answer: Thank you very much for your valuable and constructive comments. In view of the lack of detailed analysis and structured elaboration of the interaction mechanism, the author adds a new 2.2.3 section in Section 2.2 to elaborate on the specific situation of the dynamic GCT-CET interaction mechanism in this paper. At the same time, after carefully combing the structure of this paper, the incentive chart has been revised and optimized to make it conform to the incentive chart of this paper, and the innovation mechanism is clearly displayed so that the improvement content is clear and clear. The results of the modification are as follows: The specific results are as follows：

1) GCT-CET interaction mechanism description (Page 6, line 167 to line 185)

*2.2.3 Green certificate-carbon joint trading mechanism*

The integration of GCT and CET mechanisms in this work is reflected in two key aspects: (1) Carbon emission reductions from renewable energy are accounted for in the carbon quota evaluation, where renewable generation offsets part of the emission allowance[38]. (2) Dynamic reward–penalty mechanisms based on green certificate attributes guide system scheduling toward increased renewable energy utilization. Green certificates participate in both CET and GCT systems, enabling their coordinated interaction and reinforcing low-carbon dispatch objectives. The precise stages of the joint mechanism are as follows:

1) According to Eq., the carbon emissions and carbon emission quotas of IES units are calculated.

2) According to the new energy data, the carbon emission reduction caused by the green certificate is calculated according to Eq., that is :

$$E_{\text{green}}=D_{\text{carbon}}-D_{\text{green}}$$

In the formula, Egreen represents the carbon emission reduction caused by new energy power generation; Dgreen and Dcoal denote the carbon emission equivalents of new energy supply and carbon-emitting supply, respectively, over their respective periods.

3) According to the Eqs.-, calculate the new carbon emission trading amount after the new energy offset and the coefficient of rewards and punishment.

$$G_{IES,\text{new}}=\left(G_{IES,a}-D_{IES}^{\text{Total}}-E_{\text{green}}\right)$$

2) Perfecting the mechanism diagram (Page 4, line 116 to line 119)

The dynamic reward and punishment GCT-CET interaction mechanism is introduced in detail, and its structure is shown in Fig. 2.

Fig 2. Dynamic reward and punishment GCT-CET mechanism diagram

3. Section 3.2 suffers from fragmented organization. The multiple strategy modules lack a coherent design logic and are presented in isolation without clarifying their overall purpose, applicable stages, or interrelations.

Authors’ Answer: We sincerely thank the reviewer for this valuable feedback. In the revised manuscript, Section 3.2 has been restructured to clarify the overall design logic and ensure coherence among the three strategy modules. The author re-combed Section 3.2 to clarify the role of each module. At the beginning of Section 3.2, the internal and end of the MSAGOOSE algorithm framework clearly stated the overall goal, the application stage or the mutual relationship. Specifically, we emphasize that:

1）Adaptive parameter adjustment (Section 3.2.1) governs the early-stage search by dynamically tuning control parameters to set the exploration–exploitation balance.

2）Multimodal distribution guidance (Section 3.2.2) is applied during the mid-stage search to maintain population diversity and mitigate premature convergence.

3）Population-aware reverse learning (Section 3.2.3) is applied in the final stage to refine local exploitation and improve convergence accuracy.

These modules are now explicitly connected in a closed-loop logic of “parameter regulation → global exploration → local exploitation,” with each module’s applicable stage and functional role clearly stated. This restructuring improves readability, highlights the synergy among modules, and aligns the algorithm design more closely with the engineering requirements of integrated energy system scheduling. The specific details are as follows (Page 9-11, line 255 to line 318)：

***3.2 Goose algorithm improvement strategy***

This section proposes three coordinated strategies to address the key limitations of the GOOSE algorithm. Adaptive parameter adjustment (Section 3.2.1) guides global exploration, multimodal search (Section 3.2.2) enhances diversity to avoid premature convergence, and reverse learning (Section 3.2.3) improves local exploitation and convergence accuracy. Together, they form a logical closed loop of “parameter regulation → global exploration → local exploitation”, effectively balancing exploration and exploitation in high-dimensional search spaces.

*3.2.1 Adaptive adjustment factor*

The GOOSE algorithm's conditional judgment parameters are fixed and cannot be modified to accommodate diverse challenges, thereby diminishing its efficiency. The adaptive adjustment strategy is integral to the whole iterative process, focusing on balancing global exploration with local development, optimizing the optimization path, and addressing convergence instability resulting from fixed parameters.

*3.2.2 Multimodal distribution guided walk strategy*

The original GOOSE algorithm relies on a single random distribution during initial exploration, making it prone to local optima and imbalanced exploration–exploitation in high-dimensional, complex search spaces. This work introduces a Multimodal Distribution-Guided Random Walk (MDGRW) to overcome these limitations, enhancing global search robustness. MDGRW effectively balances locality and randomness, providing a generalized, extensible, and interpretable exploration framework.

*3.2.3 Population distribution-aware reverse learning strategy*

In later iterations, the traditional GOOSE algorithm suffers from degraded computational efficiency in high-dimensional IES scheduling due to boundary stagnation. Search efficiency declines exponentially with dimensionality, causing performance degradation, exploration–exploitation imbalance, and entrapment in local optima. To address this, the proposed strategy dynamically adjusts search intensity using the population mean and an attenuation factor *d*, effectively mitigating efficiency loss and controlling computational complexity as the boundary expands with dimensionality.

This paper addresses the deficiencies of the GOOSE algorithm by proposing three enhancement strategies aimed at improving its performance: the introduction of an adaptive adjustment factor for global regulation, a dynamic adjustment strategy for selecting parameter p (which governs the exploration-development strategy and MDGRW selection), and the optimization of the search range parameter d (which refines neighborhood exploration intensity). These strategies create a closed loop of ' parameter regulation→distribution exploration→neighborhood refinement ', effectively addressing the imbalance in high-dimensional space exploration and development, thereby facilitating the real-time and efficient resolution of IES scheduling cases.

4. Section 4.1 introduces CEC benchmark function testing to evaluate algorithm performance; however, the logical connection between this part and the integrated energy system scheduling problem is unclear. The testing objective appears disconnected from the main model, making this section seem isolated within the overall structure.

Authors’ Answer: We sincerely thank the reviewer for this valuable feedback.we have strengthened the explanation of the rationale for including CEC2017 and CEC2022 benchmark testing in Section 4.1 and clarified its direct connection to the IES scheduling problem.

The integrated energy system (IES) scheduling model exhibits high dimensionality, nonlinear constraints (e.g., tiered carbon pricing, green certificate trading), and tightly coupled source–grid–load–storage interactions. These complexities can obscure the direct attribution of algorithmic performance when applied solely in the IES context. To decouple algorithm behavior from model-specific intricacies, the proposed MSAGOOSE algorithm is first evaluated on the well-established CEC benchmark suites, which replicate many of the structural challenges found in IES scheduling.

The specific details are as follows (Page 12, line 326 to line 332)：

The IES scheduling model features high dimensionality, nonlinear constraints (e.g., tiered carbon pricing, green certificate trading), and tightly coupled source–grid–load–storage interactions. These complexities hinder clear attribution of algorithmic performance in direct applications. To decouple algorithm behavior from model intricacies, the proposed method is first evaluated on the CEC2017 and CEC2022 benchmark suites, which reflect similar structural characteristics[39][40]. This pre-validation offers strong quantitative evidence of the algorithm’s robustness and suitability for the real-world scheduling problem discussed in Section 5.

-------------------------------------------------------------------------------------------------------

The author is very grateful to the editors and reviewers for their prudent assessment and constructive opinions. The opinions of experts accurately identify the defects of the article, which are helpful to improve the expression of the paper and improve the quality of the research paper. Once again, we appreciate for Editors/Reviewers' warm work earnestly, and hope that the correction will meet with approval. The above reply form is also accompanied by the revised manuscript.
